# Biomarkers of ulcerative colitis disease activity CXCL1, CYP2R1, LPCAT1, and NEU4 and their relationship to immune infiltrates

**DOI:** 10.1038/s41598-023-39012-w

**Published:** 2023-07-26

**Authors:** Aijing Huo, Fengmei Wang

**Affiliations:** 1 Department of Nephropathy and Immunology, The Third Central Clinical College of Tianjin Medical University, No. 83 Jintang Road, Hedong District, Tianjin, 300170 China; 2 Tianjin Key Laboratory of Extracorporeal Life Support for Critical Diseases, Artificial Cell Engineering Technology Research Center, Tianjin Institute of Hepatobiliary Disease, The Third Central Hospital of Tianjin, Tianjin, China; 3 Department of Gastroenterology and Hepatology, The Third Central Clinical College of Tianjin Medical University, No. 83 Jintang Road, Hedong District, Tianjin, 300170 China

**Keywords:** Data mining, Inflammatory bowel disease

## Abstract

The diagnosis and assessment of ulcerative colitis (UC) poses significant challenges, which may result in inadequate treatment and a poor prognosis for patients. This study aims to identify potential activity biomarkers for UC and investigate the role of infiltrating immune cells in the disease. To perform gene set enrichment analysis, we utilized the cluster profiler and ggplot2 packages. Kyoto encyclopedia of genes and genomes was used to analyze degenerate enrichment genes. Significant gene set enrichment was determined using the cluster profiler and ggplot2 packages. Additionally, quantitative PCR (qRT-PCR) was employed to validate the expression of each marker in the ulcerative colitis model. We identified 651 differentially expressed genes (DEGs) and further investigated potential UC activity biomarkers. Our analysis revealed that CXCL1 (AUC = 0.710), CYP2R1 (AUC = 0.863), LPCAT1 (AUC = 0.783), and NEU4 (AUC = 0.833) were promising activity markers for the diagnosis of UC. Using rat DSS model, we validated these markers through qRT-PCR, which showed statistically significant differences between UC and normal colon mucosa. Infiltrating immune cell analysis indicated that M1 macrophages, M2 macrophages, activated dendritic cells (DCs), and neutrophils played crucial roles in the occurrence and progression of UC. Moreover, the activity markers exhibited varying degrees of correlation with activated memory CD4 T cells, M0 macrophages, T follicular helper cells, memory B cells, and activated DCs. The potential diagnostic genes for UC activity, such as CXCL1, CYP2R1, LPCAT1, and NEU4, as well as the infiltration of immune cells, may contribute to the pathogenesis and progression of UC.

## Introduction

Ulcerative colitis (UC) is classified as an inflammatory bowel disease (IBD)^1^. In patients with UC, the cumulative inflammatory burden is strongly associated with the risk of colorectal neoplasia^2^. Given its autoimmune nature, UC is known to be influenced by immune factors^3^.

Recent studies have suggested that immune cell infiltration plays a significant role in the development of ulcerative colitis. Alterations in M1/M2 macrophages can impact various inflammatory reactions, leading to persistent inflammation in and around the necrotic area of the colon^4^. Activation of dendritic cells (DCs) and neutrophils has also been linked to an increased incidence of colitis^5,6^.

Several immune-related factors have been linked to ulcerative colitis, including increases in colonic regulatory T cells with enhanced expression of the transcription factor ZEB2, increased IgG1 plasma cells, and enrichment of gamma-T cell subpopulations in peripheral blood^7^. Moreover, different phases of development of CD8 tissue resident memory T (T.RM) cells in the colon tissue have been associated with the potential development of UC. The interleukin (IL)- 23/IL-17 axis has also been shown to play a significant role in the pathogenesis of UC by promoting Th17 cells and cytokine-related immunological responses^8^.

Assessing the degree of immune cell infiltration and differentiation of infiltrating immune cells is crucial for developing new immunotherapeutic targets and understanding the molecular pathology of UC. The CIBERSORT algorithm, based on gene expression, can be used to identify immune cells and pathways. Previous studies have demonstrated the combination of GEO and CIBERSORT in a variety of complex diseases such as sepsis^9^, osteoarthritis^10^ and steroid-induced osteonecrosis of the femoral head^11^. Additionally, single sample gene set enrichment analysis (ssGSEA) can be employed to identify significant immune cells and pathways^12^. The immune system and inflammation play significant roles in numerous disease processes^13^. However, few studies have utilized both CIBERSORT and ssGSEA to evaluate immune cell infiltration in UC and its potential clinical value.

In this study, we utilized machine learning algorithms and various bioinformatics approaches to identify microarray-based diagnostics of UC downloaded from the gene expression omnibus (GEO) database (http://www.ncbi.nlm.nih.gov/geo/). Furthermore, we employed CIBERSORT and ssGSEA to compare 22 immune cell subsets infiltrating normal and ulcerative colitis tissues. Our objective was to gain a deeper understanding of the underlying molecular immunological mechanisms involved in UC development and to determine whether immune cell infiltration and activity biomarkers are correlated.

## Materials and methods

### Data collection

We conducted a search for datasets related to “ulcerative colitis” in the GEO database 1 using the search terms “Homo sapiens” and “Expression profiling by array”. Microarray datasets containing human peripheral serum gene expression profiles were selected, which included both normal and UC colon mucosa. We selected GSE48634, GSE107499, and GSE179285 for further investigation. All colon mucosa samples included in our analysis had a diagnosis of UC and were collected from mucosal biopsies (n = 368) located 15–20 cm from the anal verge under endoscopic screening. Crohn’s disease samples were excluded from our analysis. Finally, we used GSE92415 for subsequent analysis of 162 UC samples and 21 healthy controls. The healthy controls were recruited during the clinical trial and showed no macroscopic evidence of mucosal pathology.

### Data

#### DEGs preprocessing and identification

To preprocess the initial data, we utilized the robust multiarray averaging (RMA) method with the “affy” package’s background correction and normalization procedure^14^. Subsequently, the limma package was employed in R software to identify differentially expressed genes (DEGs) between UC and healthy controls (HCs)^15^. The *P* values were adjusted using the Benjamini and Hochberg test, and the significance was determined using a cut off criteria of *P* < 0.05 and |log2FC| > 1.

#### Functional correlation analysis

To perform functional annotation of the DEGs, we conducted gene ontology (GO) and Kyoto encyclopedia of genes and genomes (KEGG) enrichment analyses using the R packages “cluster Profiler” and “ggplot2”. For KEGG pathway analysis, the degenerate enrichment genes were analyzed using the KEGG package^16–18^. Moreover, we utilized the “cluster Profiler” and “ggplot2” packages to perform gene set enrichment analysis (GSEA) on the gene expression matrix with a significance threshold of *P* < 0.05.

### Diagnostic screening and verification

#### Markers

To screen diagnostic biomarkers for UC activity, we used logistic regression with the least absolute shrinkage and selection operator (LASSO)^19^, support vector machine-recursive feature elimination (SVM-RFE)^20^, and random forests (RF)^21^. We implemented the LASSO method using the “glmnet” package^22^, while SVM-RFE, a support vector machine-based machine learning technique, was used to identify the most advantageous variables by eliminating eigenvectors produced by SVMs. The SVM function included in the R-software e1071 package was utilized to implement the SVM model^23^. For the random forest (RF) technique, randomized algorithm techniques were used to increase accuracy from a large number of pertinent decision trees from a single training set of trees while reducing the overfitting of a single decision tree^24^. These three classification model algorithm genes were retrieved and used for further investigation. We tested CXCL1, CYP2R1, LPCAT1, and NEU4 in-depth for their diagnostic abilities using receiver operating characteristic (ROC) curves in Med Calc software. The area under the curve (AUC) was calculated as the critical diagnosis-related index, and a* P* < 0.05 demonstrated statistical significance.

#### Analysis of quantitative PCR

To validate the prediction results further, we performed quantitative reverse transcription PCR (qRT-PCR) to investigate the expression of the four activity markers that were found to be linked to abnormal expression in the colon of dextran sulfate sodium (DSS) rats.

SPF-grade male Sprague–Dawley (SD) rats (230–280 g, 6–8 weeks of age, Beijing Huafukang) were cultured in the animal laboratory of Nankai Hospital in Tianjin. Rats were kept in a research environment under the following guidelines: They had seven days to get used to the conditions—25 °C ± 3 °C, 53 ± 3% humidity, a 12-h light/dark cycle—while having unrestricted access to food and water. The DSS-induced UC model has immunological characteristics similar to those of human UC^25,26^. Since DSS-induced murine models display patho-morphological changes similar to human ulcerative colitis, they have often been used in experimental studies^27,28^. 24 rats were randomly divided into two groups: 15 rats were DSS colitis models (DSS group) and 9 rats were controls (HC group). DSS model was made with reference to previous experiments^29^. 3% (wt/vol) dextran sulfate sodium (D808272, DSS, macklin, China) was added to the drinking water of DSS group for 12 weeks. SD rats were deeply anesthetized by sodium pentobarbital and colon specimens were isolated and immediately snap-frozen in liquid nitrogen. All tissues were stored at − 80 °C until analysis. This study was approved by the Animal Care and Ethics Committee of Tianjin Nankai Hospital (SYXK2020-0008) for animal experiments, which was developed based on the Helsinki convention for the use and care of animals and reported in consent with the ARRIVE guidelines. All efforts were made to minimize animal suffering and reduce the number of animals used.

RNA extraction was performed on all samples using TRIzol reagent (TAKARA, Dalian, China). Subsequently, cDNA was synthesized using a cDNA reverse transcription kit (Applied Biosystems) and amplified with a SYBR Green PCR kit (Qiagen Germany). The internal reference GAPDH was used to normalize the data. The amplification reaction was carried out in 20 μl volumes under the following conditions: initial denaturation (95 °C, 2 min), followed by 40 cycles of denaturation (95 °C, 30 s), annealing (58 °C, 30 s), extension (72 °C, 30 s), and a final cooling step (40 °C, 30 s). The 2-ΔΔCt method was used to calculate the relative mRNA expression. The primers used in the reverse transcription PCR are listed in Table 1.

**Table 1:** List of RT–PCR primer sequences.

Target genes	Sequence
CXCL1-RAT-qF	ACCGAAGTCATAGCCACACT
CXCL1-RAT-qR	GGGACACCCTTTAGCATCTT
CYP2R1-RAT-qF	GAGCGATTTCTGGACAGCAG
CYP2R1-RAT-qR	AGTTCGTGTGGGAAATGCAA
LPCAT1-RAT-qF	GCATCCTCAAGACTGCACTG
LPCAT1-RAT-qR	CTCTGCGAAGTCAGGGTACA
NEU4-RAT-qF	TTTGCCTGCCTGTTTGAGAG
NEU4-RAT-qR	AGCCAGTGGGTACATTCTCC
GAPDH-RAT-qF	CAAGGCTGAGAATGGGAAGC
GAPDH-RAT-qR	GAAGACGCCAGTAGACTCCA

### Evaluation of immune cell infiltration

We utilized CIBERSORT^30^ and ssGSEA scores^31^ to transform the gene expression matrix into an immune cell matrix and integrated it with the composition and percentages of the immune cell matrix. The immune cell matrix was generated by employing the gene expression matrix and the immune cell composition and percentages, and samples with a *P* value of less than 0.05 underwent filtration to obtain the immune cell matrix. Subsequently, we performed PCA clustering analysis on the immune cell infiltration matrix data using the “ggplot2” software, which enabled us to create a 2D PCA clustering map. The use of principal component analysis (PCA) as a multidimensional scaling method is widely accepted^32^. PCA was applied to identify the underlying causes of the correlation pattern within a set of observed variables in the normal and UC groups. We created a correlation heatmap using the R package “corrplot” to display the connections between the 22 different types of immune cells associated with infiltration^33^. Heatmap diagrams were constructed using the “ggplot2” software to visualize the differences in immune cell infiltration. Statistical significance was defined as *P* values < 0.05.

### Identification of genes and intration-related immune cells

The relationship between the quantity of infiltration-related immune cells and the identified gene biological indicators was evaluated through Spearman's rank correlation analysis using the R programming language. The “ggplot2” package was utilized to create visual representations of the correlations. A *P* value of less than 0.05 was considered statistically significant.

## Results

### Identifying DEGs and preprocessing data

**Figure 1:**
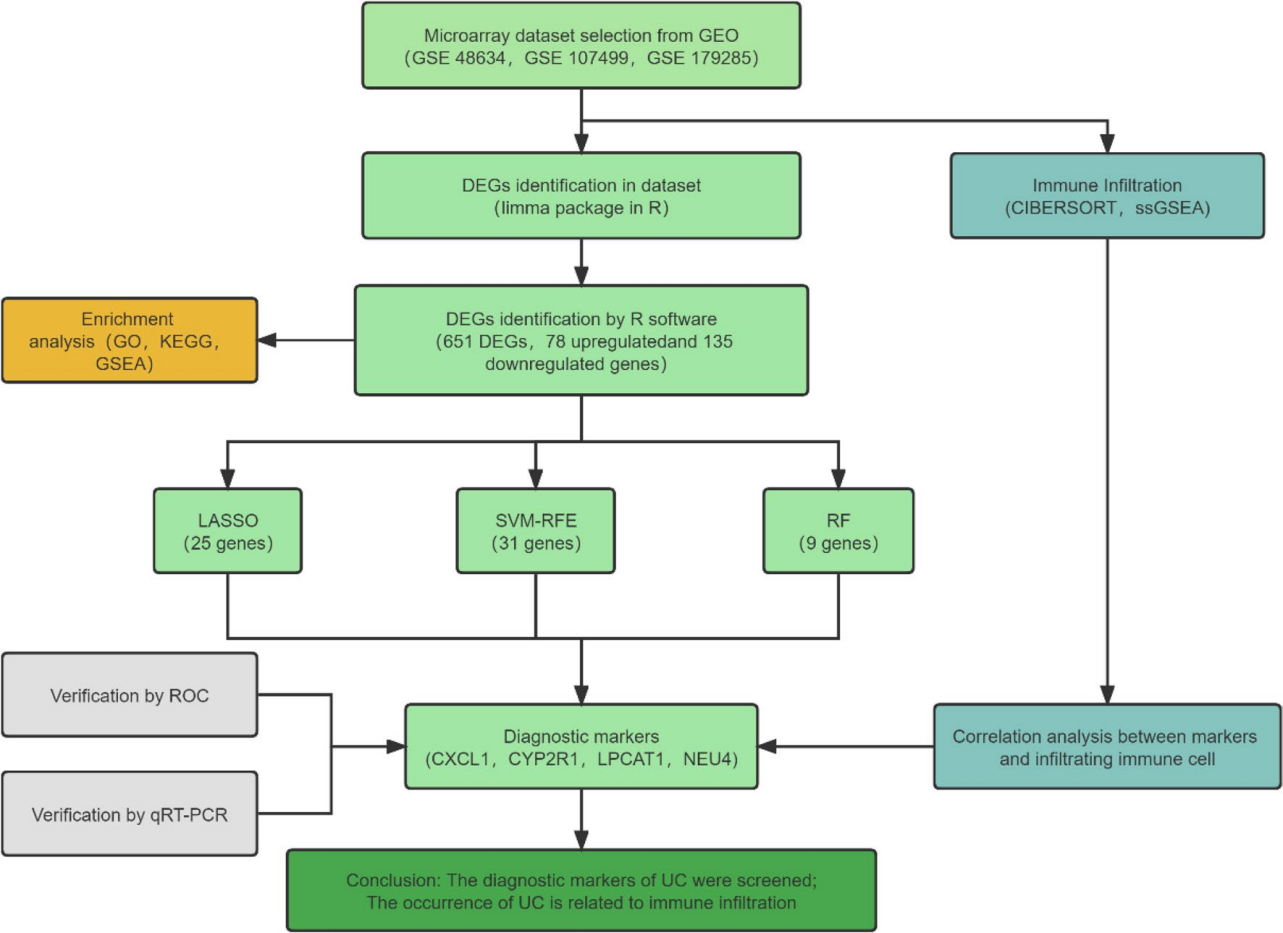
Process flow diagram for the analysis.

**Figure 2:**
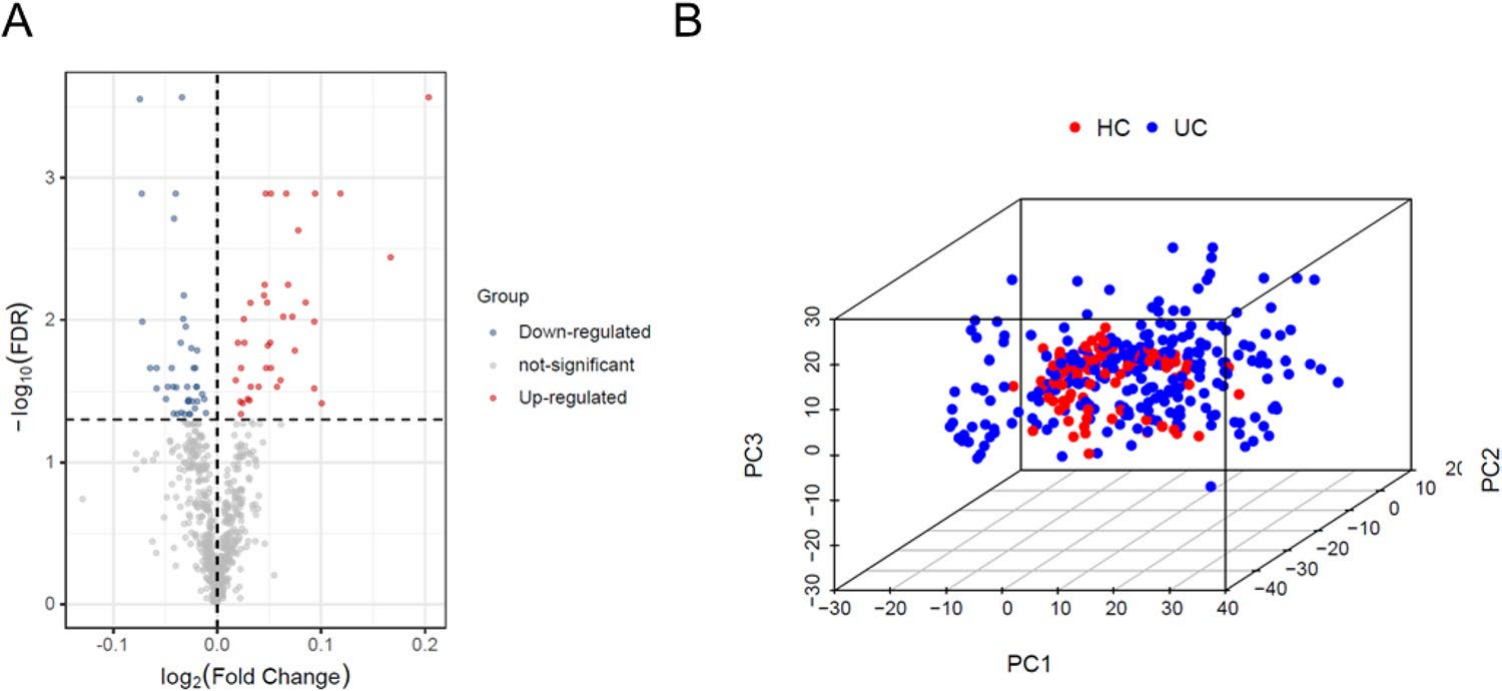
Data preprocessing and DEG screening. (**A**) A volcano map of differentially expressed genes shows that red genes are upregulated, black genes are not significantly different, and blue genes are downregulated. (**B**) PCA 3D plot, differentially expressed genes between the HC and UC groups; red represents the HC group, and blue represents the UC group.

Figure 1 shows the workflow used in this study. Firstly, the differentially expressed genes (DEGs) were identified from the gene expression matrix using R software. A total of 651 DEGs were extracted, of which 135 were downregulated and 78 were upregulated (Supplementary List 1). Figure 2A and B provide a volcano plot and heatmap, respectively, displaying the distribution of DEGs.

### Functional enrichment analyses

**Figure 3:**
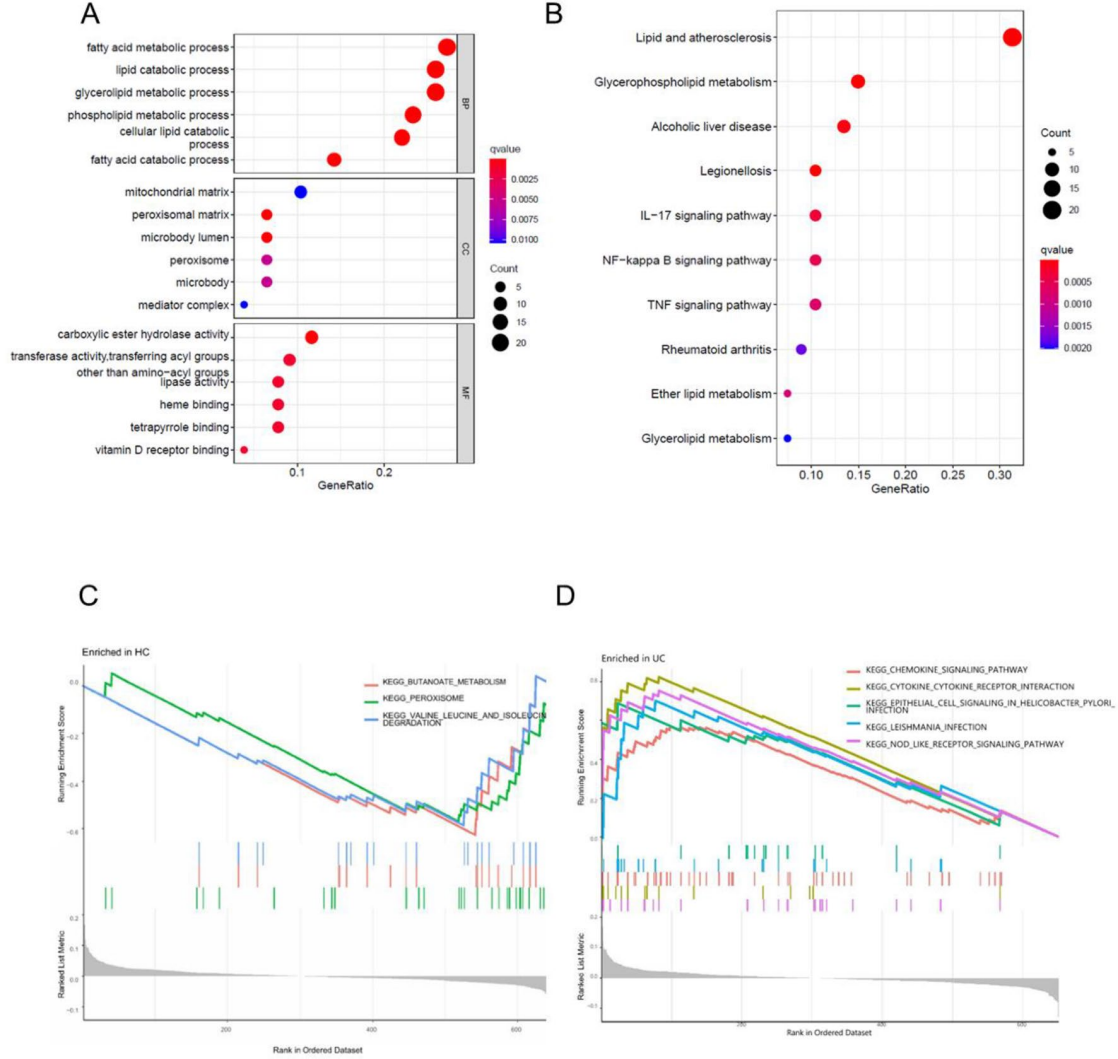
Functional enrichment analysis results. (**A**) Functional enrichment analysis results. (**B**) Results of DEG pathway analysis; (**C**) GSEA results indicating the most related signaling pathways to HCs; (**D**) GSEA results showing that the top 5 signaling pathways are most associated with UC.

The differentially expressed genes (DEGs) identified in this study were further analyzed using gene ontology (GO) analysis to identify their biological functions. The results showed that the DEGs were mainly associated with fatty acid metabolism, lipid catabolic processes, glycerolipid metabolic processes, and phospholipid metabolic processes that were involved in the immune response (Fig. 3A). Additionally, the DEGs were enriched in 10 pathways according to KEGG pathway analysis (Fig. 3B). The gene set enrichment analysis (GSEA) demonstrated that the normal group was primarily enriched in butanoate metabolism and peroxisome pathways, while the main pathways enriched in the UC group were related to chemokine signaling, cytokine receptor interactions, signaling in helicobacter pylori infection, leishmania infection, and NOD-like receptor signaling (Fig. 3C,D). These findings were confirmed by KEGG pathway analysis.

### Identifying and verifying activity biomarkers

**Figure 4:**
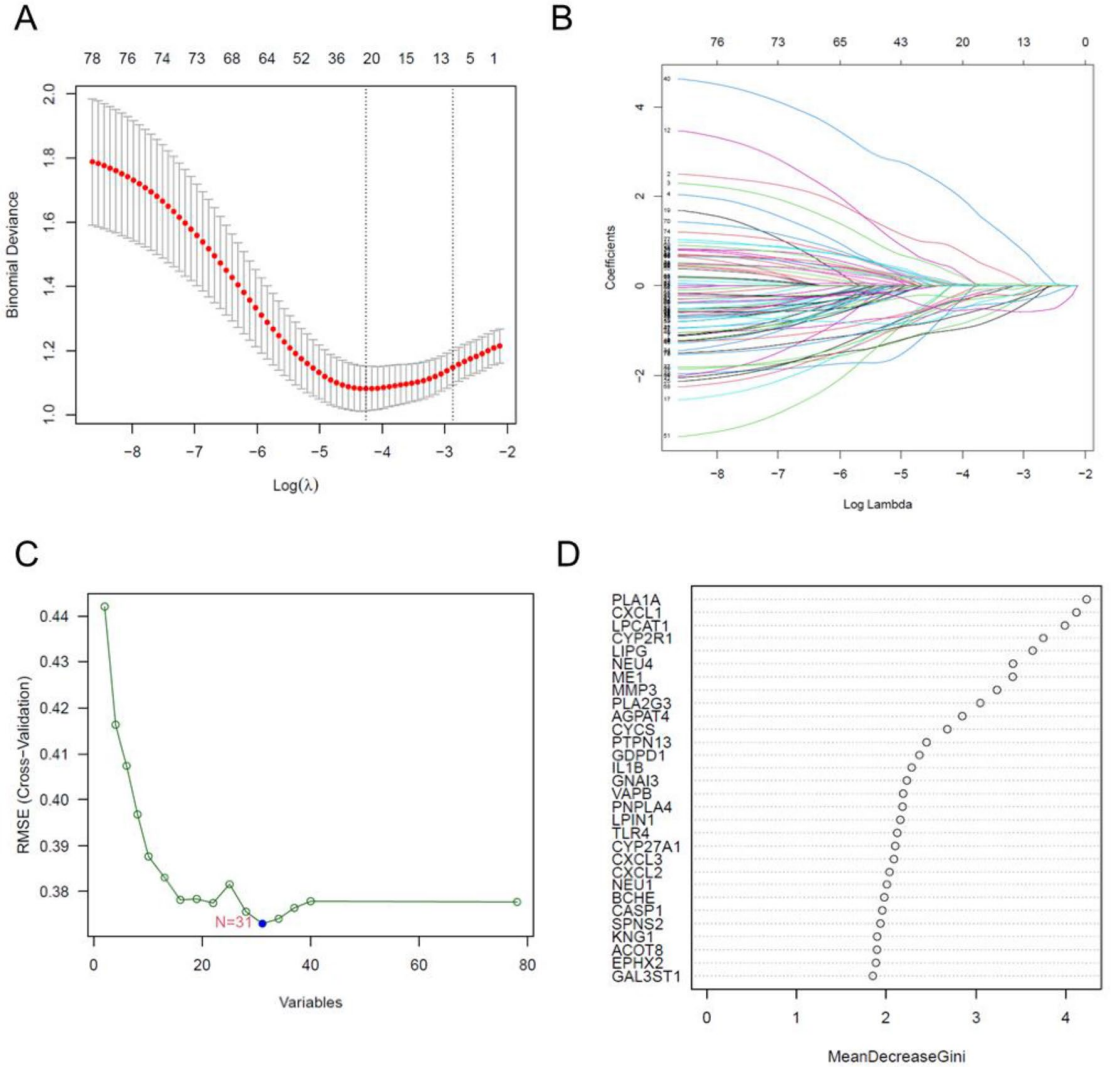
Diagnostic indicators are screened using a thorough methodology. (**A**,**B**) The varied colors represent distinct genes when using the LASSO technique to screen diagnostic markers; (**C**,**D**) recursive feature elimination (SVM-RFE) and random forest (RF) algorithms are employed to screen biomarkers.

The present study utilized various bioinformatics tools to identify potential diagnostic markers for ulcerative colitis (UC) based on the analysis of differentially expressed genes (DEGs) (Fig. 4A). Gene ontology (GO) and KEGG pathway analyses revealed that the DEGs were mainly associated with lipid metabolic processes and immune responses. Using LASSO logistic regression, SVM-RFE, and RF algorithms, 25, 31, and 9 DEGs, respectively, were identified as possible diagnostic activity markers for UC (Fig. 4B–D).

**Figure 5:**
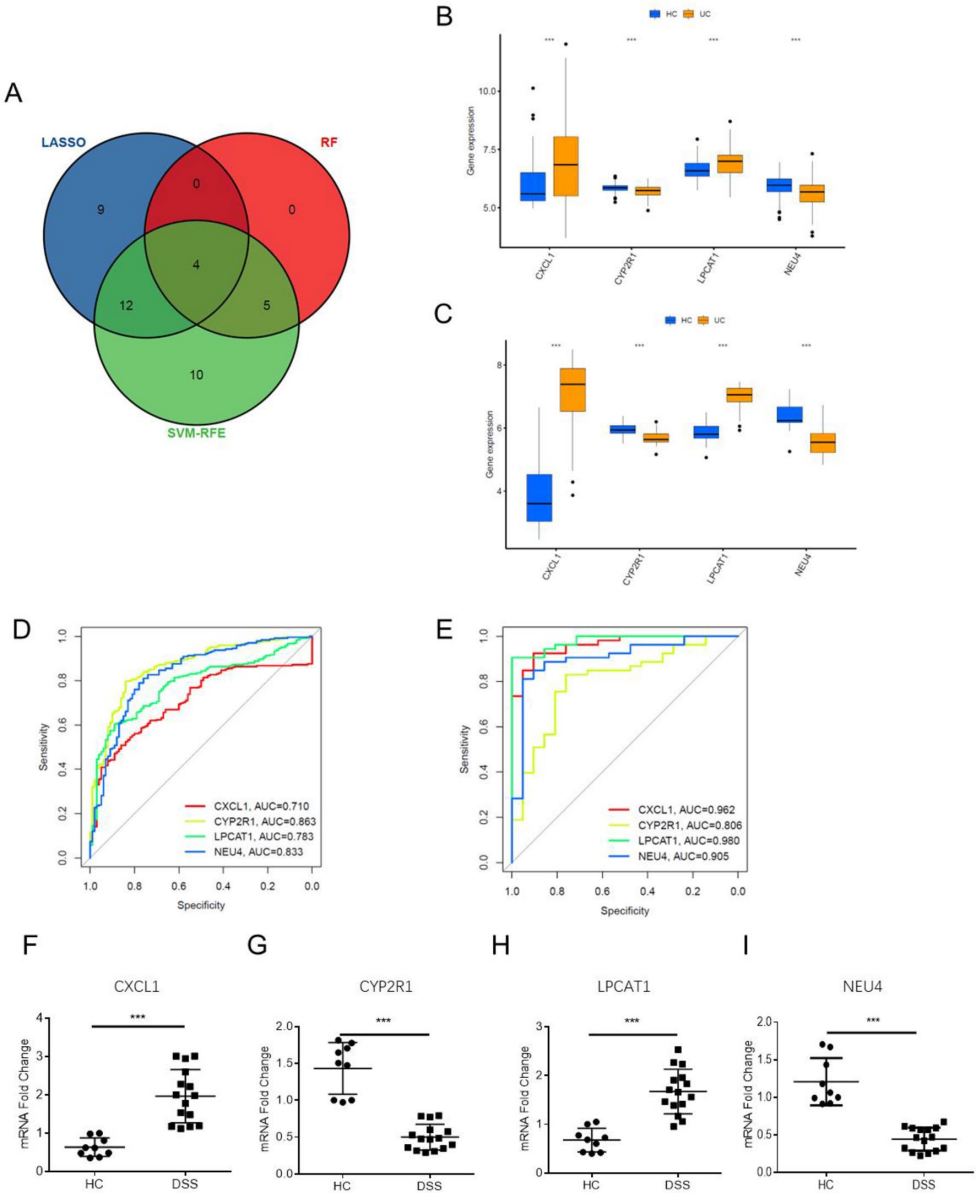
(**A**) The intersection of the diagnostic markers that the three algorithms produced is depicted by a Venn diagram. (**B**) Boxplot of hub genes in the training group; (**C**) Boxplot of hub genes in the test group. (**D**) The ROC curve of the diagnostic efficacy in the training group; (**E**) the ROC curve of the diagnostic efficacy in the test group, verification after fitting four diagnostic activity biomarkers to one variable. (**F**–**I**) qRT‒PCR was used to determine the expression of potential diagnostic markers (n = 24). *P < 0.05; **P < 0.01, ***P < 0.001.

Four diagnostic markers were ultimately selected based on the overlapping marker genes obtained from the three algorithms (Fig. 5A). The accuracy of the four diagnostic markers was verified using test samples, and their high diagnostic value was confirmed through the analysis of ROC curves. To validate the results from the training cohort, a test cohort consisting of different samples was generated, and the diagnostic markers' utility was again demonstrated. These findings suggest that the four diagnostic markers (CXCL1, CYP2R1, LPCAT1, and NEU4) have the potential to be useful in diagnosing UC activity. Moreover, qRT-PCR results further support the differential expression of these markers in UC tissues. The diagnostic potential of CXCL1, CYP2R1, LPCAT1, and NEU4 expression as determined by the ROC curves was further confirmed in the test cohort, with the calculated AUC values of 0.962, 0.806, 0.980, and 0.905, respectively (Fig. 5B–E).

The mRNA expression levels of these diagnostic markers were assessed using qRT-PCR, as illustrated in Fig. 5F–I. In total, 15 colon tissues and 9 UC tissues were obtained, and a DSS colitis model was established according to previously reported methods^29^. Statistical significance was detected for all four diagnostic markers (*P* < 0.05). Notably, NEU4 and CYP2R1 were downregulated in the UC group compared to the HC group, while CXCL1 and LPCTA1 exhibited higher levels in the UC group than in HC participants. These results indicate the potential clinical value of the identified diagnostic markers in UC detection and management.

### Infiltration of immune cells

**Figure 6:**
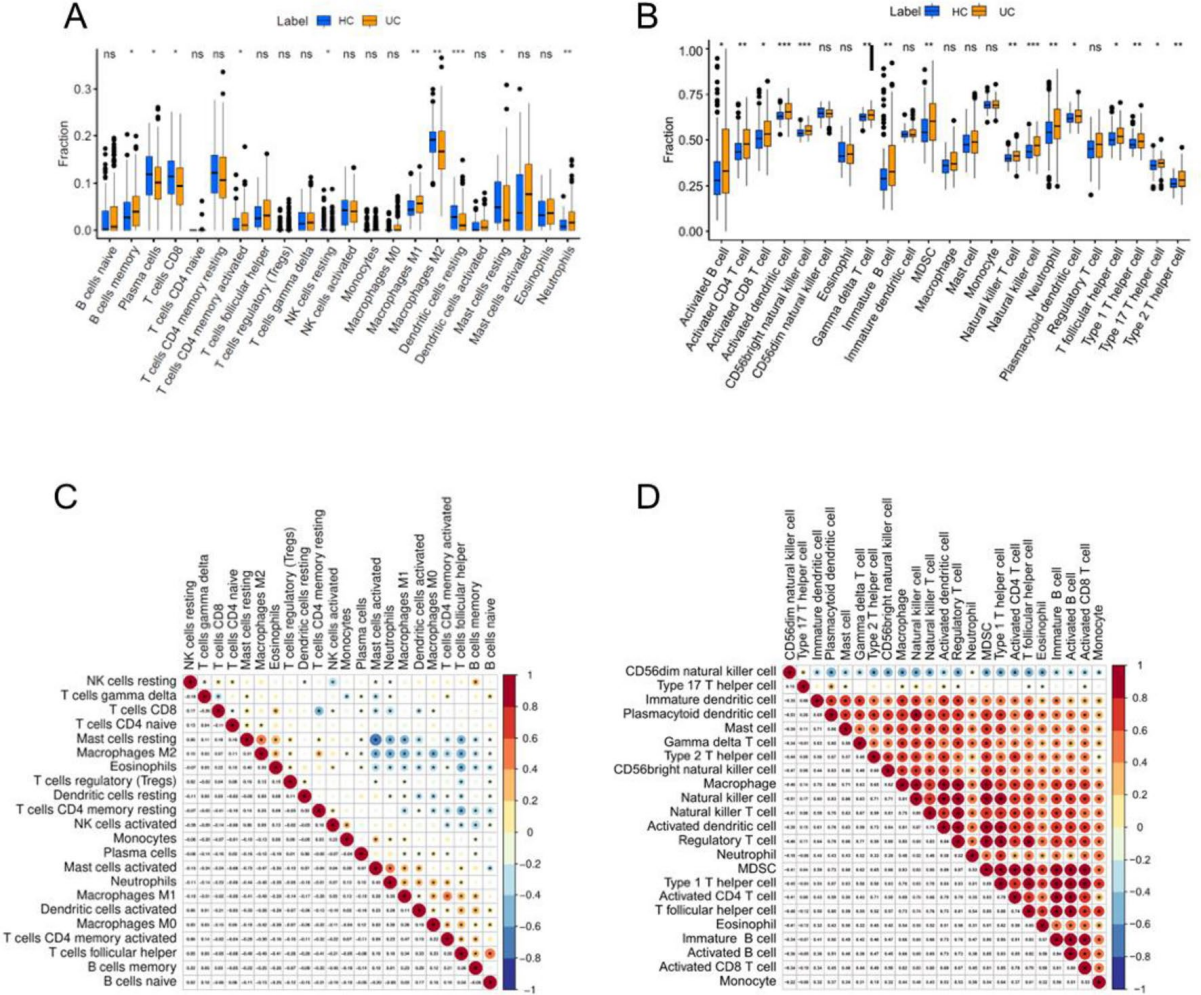
Immune cell infiltration assessment and visualization. (**A**) Using the CIBERSORT R program, a boxplot of the proportions of 22 different types of immune cells is shown. (**B**) Boxplot showing the distribution of 22 different immune cell types based on ssGSEA scores. (**C**) CIBERSORT R software extracted the correlation coefficient of 22 different immune cell types and eliminated the correlation coefficient of one immune cell type without affecting the results. (**D**) The correlation coefficient was adjusted to exclude one type with no difference and 22 types of immune cells with ssGSEA scores. *P < 0.05; **P < 0.01, ***P < 0.001.

The levels of infiltrating immune cells in UC and normal tissues were assessed using two different methods, CIBERSORT and ssGSEA, encompassing a total of 22 different immune cell types. CIBERSORT analysis revealed significant differences in immune cell infiltration between the two groups (Fig. 6A). Specifically, UC tissues exhibited lower levels of infiltrating M2 macrophages, resting dendritic cells, CD8 T cells, and plasma cells, whereas higher levels of infiltrating memory B cells, M1 macrophages, and neutrophils were observed compared to normal samples. ssGSEA analysis also revealed a substantial difference in the numbers of invading immune cells between the two groups (Fig. 6B). In UC tissues, lower levels of infiltrating T helper (TH) 1 cells and plasmacytoid dendritic cells (DCs) were observed, whereas higher levels of infiltrating activated B cells, activated CD4 T cells, activated CD8 T cells, DCs, CD56-bright natural killer cells, gamma delta T cells, immature B cells, myeloid-derived suppressor cells (MDSCs), natural killer T cells, natural killer cells, neutrophils, T follicular helper cells, TH17 cells, and TH2 cells were observed compared to normal tissues.

Our analysis using CIBERSORT also revealed multiple pairs of immune cells that were positively or negatively associated with each other (Fig. 6C). The correlation score was calculated to indicate the strength of the association. We found significant negative correlations between resting mast cells and neutrophils, resting mast cells and active mast cells, resting mast cells and T follicular helper cells, and T follicular helper cells and resting memory CD4 T cells. On the other hand, we observed significant positive correlations between naive B cells and T follicular helper cells, activated memory CD4 T cells and M1 macrophages, activated mast cells and neutrophils, resting mast cells and M2 macrophages, and eosinophils and CD8 T cells. These findings provide insights into the complex interactions between different immune cell types in UC.

According to the results obtained from ssGSEA (Fig. 6D), multiple pairs of immune cells were found to be either positively or negatively correlated, and their correlation strength was expressed as a score. Immature DCs, plasmacytoid DCs, mast cells, gamma delta T cells, TH2 cells, CD56-bright natural killer cells, macrophages, natural killer cells, natural killer T cells, activated DCs, regulatory T cells, neutrophils, MDSCs, TH1 cells, activated CD4 T cells, T follicular helper cells, eosinophils, immature B cells, activated B cells, activated CD8 T cells, monocytes, and CD56-dim NK cells were significantly correlated with each other.

### An examination of the relationship between the expression of activity biomarkers and the numbers of infiltrating immune cells

**Figure 7:**
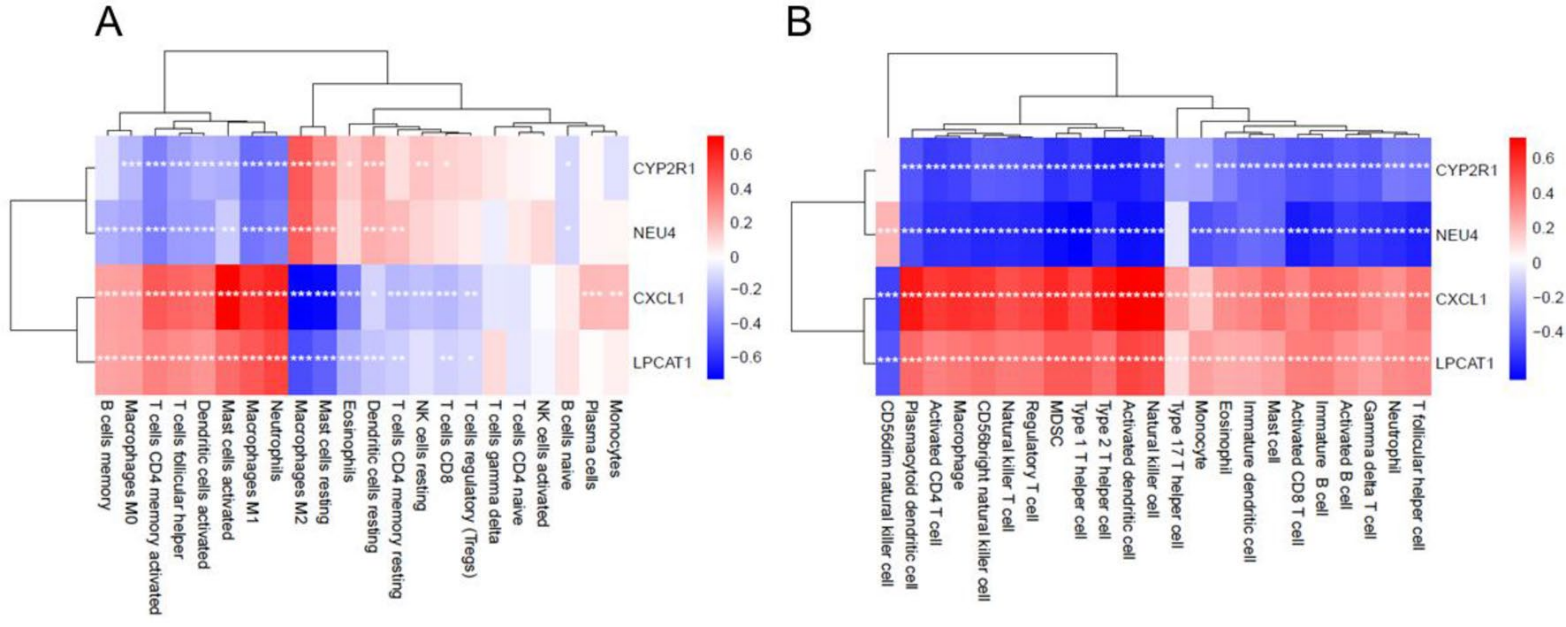
Correlation between activity biomarkers and infiltrating immune cells. (**A**) Twenty-two types of immune cells of ssGSEA scores and one type with no difference were removed from the correlation coefficient. (**B**) ssGSEA results are correlated with invading immune cells and activity biomarkers. Red indicates a positive association, whereas blue indicates a negative correlation in the form of colored squares. *P < 0.05; **P < 0.01, ***P < 0.001.

As shown in Fig. 7A, correlation analysis based on CIBERSORT revealed that CXCL1 expression was positively correlated with memory B cells, M0 macrophages, activated memory CD4 T cells, T follicular helper cells, activated DCs, activated mast cells, M1 macrophages, plasma cells, monocytes, and neutrophils, while it was negatively correlated with M2 macrophages, resting mast cells, eosinophils, resting memory CD4 T cells, resting NK cells, and CD8 T cells. Furthermore, LPCAT1 expression was positively correlated with memory B cells, M0 macrophages, T follicular helper cells, activated memory CD4 T cells, activated DCs, activated mast cells, M1 macrophages, and neutrophils, while it was negatively correlated with M2 macrophages, resting mast cells, eosinophils, and resting DCs. Moreover, CYP2R1 expression was positively correlated with M0 macrophages, T follicular helper cells, activated memory CD4 T cells, activated DCs, activated mast cells, M1 macrophages, and neutrophils, and positively correlated with M2 macrophages, resting mast cells, and resting DCs. Finally, NEU4 expression was negatively correlated with memory B cells, M0 macrophages, activated memory CD4 T cells, T follicular helper cells, activated DCs, M1 macrophages, and neutrophils, while it was negatively correlated with M2 macrophages, resting mast cells, and resting DCs.

The results from the ssGSEA analysis indicated a negative correlation between the expression of CYP2R1 and NEU4 and the levels of the majority of immune cells, as illustrated in Fig. 7B. On the other hand, the expression of NEU4 showed a positive correlation with the amounts of CD56-dim natural killer cells. Conversely, CXCL1 and LPCAT1 expression was found to be positively correlated with the levels of most immune cells, but negatively correlated with the level of CD56-dim natural killer cells. These findings suggest that CYP2R1, NEU4, CXCL1, and LPCAT1 may play important roles in the regulation of immune cell functions and their interplay in the tumor microenvironment.

It is evident from the analysis of immune cell infiltration assessment and its linkage with diagnostic markers that, out of 22 immune cells, neutrophils, M1 and M2 macrophages and activated DCs interact with other immune cells most strongly. ssGSEA scores also verified that four diagnostic markers were closely related to 22 kinds of infiltrating immune cells in the occurrence of UC.

## Discussion

Since immune cells play an important role in ulcerative colitis diagnosis and pathogenesis, it is necessary to further study the relationship of these biomarkers to the immune response of UC patient. It is essential to analyze the infiltration patterns of immune cells for the improvement of outcomes. In this study, we successfully identified UC-specific potential activity markers and further explored the association between these markers and the levels of immune cell infiltration in UC.

The GO analysis revealed a significant association between UC development and the immune system, with 651 DEGs identified. The enriched GO terms were mainly involved in immune response-related processes, including fatty acid metabolic processes, lipid catabolic processes, glycerolipid metabolic processes, and phospholipid metabolic processes. The inflamed tissues of UC patients showed noticeable alterations in bacterial functional pathways, including reduced glucose metabolism and activated lipid and amino acid metabolism^34^.

Emerging evidence suggests that fatty acid metabolism plays a crucial role in regulating immune cell differentiation and function. The pathogenesis of ulcerative colitis (UC) involves a multitude of immune cells, and fatty acid metabolism (FAM) plays a crucial role in immune cell proliferation and function. In a study on the link between FAM and colonic tissue inflammation, immune-related DEGs were significantly enriched in neutrophil migration and positively impacted T cell activation^35^.

CXCL1 is a potent neutrophil chemoattractant secreted by astrocytes^36^. Previous studies have shown a positive correlation between IL-17 levels and UC severity^37^. Upregulation of IL-17 in parenchymal cells induces recruitment of polymorphonuclear cells to the site of infection through CXCL1-mediated pathways^38^. The neutrophil response to mast cells and macrophages to be CXCL1/CXCL2-dependent^39^. Previous research has supported CXCL1 regulated by NF-κB in active UC^40^.

CYP2R1 is the enzyme responsible for the critical first step in vitamin D metabolism^41,42^. Activated vitamin D stimulates the production by neutrophils, macrophages, and cells lining epithelial surfaces of antibacterial peptides with broad antimicrobial activity^43–45^. Expression of CYP2R1 correlated with VDR expression on the inflammatory infiltrate^46^. Cells of the immune systems, such as macrophages, dendritic cells, monocytes, and T and B cell express VDR^47^.

NEU4 belongs to the group of lysosomal neuraminidases, which are enzymes that catalyze the cleavage of sialic acids linked to glycoconjugates^48^. In addition, NEU4 is the only sialidase that efficiently acts on mucins and is down-regulated in colon cancer^49^. As a negative regulator, NEU4 plays an important and unique role in regulating the migration of immune cells to inflammatory sites and subsequent inflammation. NEU4 participates in leukocyte recruitment, such as MO, NE, and NK cells, which migrate to and recruit to inflammatory sites^50^.

Previous studies have linked LPCAT1 activity to various inflammatory diseases. It is LPCAT, by controlling the physical state of the lipid micro environment in the rafts, could modulate the signalling receptor response to LPS^51^. LPC, which is reacylated by LPCAT, contributes to inflammation by increasing chemokine production and activating endothelium, neutrophils, monocytes, macrophages, and lymphocytes^52^. LPCAT may mediate the priming reactions of monocytes to the cytokine Interferon-γ^53^.

IBD pathogenesis can be augmented by inappropriate macrophage and DC responses to the microbiota^54^. These responses involve inadequate protection and strengthen pathogenicity^55^.

Macrophages are core effector cells of the innate immune system. Macrophages are functionally plastic and differentiate into pro-inflammatory (M1-like) or anti-inflammatory (M2-like) phenotypes in response to different stimuli in the local microenvironment^56^. M1 macrophages secret pro-inflammatory cytokines like TNF-α and Interferon-γ, causing mucosal damage and exacerbating inflammation. In contrast, M2 macrophages promote tissue repair and inhibit inflammatory response to alleviate IBD symptoms^57^.

Patients with UC have been found to have a decreased percentage of CD4 + CD25 + CD127-lowFoxp3 + regulatory T cells, which play a crucial role in maintaining immune tolerance^58^. Conversely, the lamina propria of UC patients has a higher number of Th17 cells, which are pro-inflammatory^59^. The interaction between TH cells and regulatory T cells is crucial for inducing and maintaining immune tolerance, and the imbalance between these two types of cells in UC can lead to abnormal immune responses^60^. Dendritic cells (DCs) are antigen-presenting cells that are essential for the adaptive immune system. T cells and DCs interact to initiate and control lymphocyte responses^61^.

The study's results indicate significant negative correlations between resting mast cells and neutrophils, resting mast cells and active mast cells, resting mast cells and T follicular helper cells, and resting memory CD4 T cells and T follicular helper cells. On the other hand, the study found significant positive correlations between naive B cells and T follicular helper cells, activated memory resting CD4 T cells and M1 macrophages, activated mast cells and neutrophils, resting mast cells and M2 macrophages, and eosinophils and CD8 T cells.

This study found correlations between the levels of infiltrating immune cells in UC. Further research and clarification are needed to better understand the complex interactions.

In the present study, advanced techniques such as RF, LASSO logistic regression, and SVM-RFE were employed to identify potential activity biomarkers for UC. Additionally, differences in immune cell infiltration between healthy tissues and UC tissues were evaluated using CIBERSORT and ssGSEA. However, the study had some limitations. Firstly, the retrospective nature of the study and the limited sample size may have skewed the results. Therefore, further validation of the findings in a larger patient cohort is warranted. Moreover, the CIBERSORT analysis was limited to genetic information from a small number of individuals and could not account for heterotypic interactions between cells, phenotypic plasticity, or disease-induced disorders. As CIBERSORT is an emerging technology, its performance in the context of UC needs to be verified. In conclusion, the findings of this study should be validated in a larger external patient cohort before they can be applied clinically.

## Conclusion

Based on the findings of our study, it can be inferred that the CXCL1 gene, as well as CYP2R1, LPCAT1, and NEU4, may serve as potential diagnostic biomarkers for UC activity. In addition, our results revealed that neutrophils, M1 and M2 macrophages, and dendritic cells play significant roles in UC pathogenesis. Furthermore, our study found a strong correlation between the levels of immune cell infiltration and the expression of the above-mentioned genes. This information may facilitate the development of targeted immunotherapies and optimization of UC treatment.

## Supplementary Information

Supplementary Information.

## Data Availability

The datasets used and/or analyzed during the current study available from the corresponding author on reasonable request.
