# Supplementary material for: Biomarkers of ulcerative colitis disease activity CXCL1, CYP2R1, LPCAT1, and NEU4 and their relationship to immune infiltrates
Source: Sci Rep. 2023 Jul 26;13:12126. doi: 10.1038/s41598-023-39012-w (PMC10372061; doi:10.1038/s41598-023-39012-w)
Supplement: Supplementary file 1 — Supplementary Information. [file 41598_2023_39012_MOESM1_ESM.pdf]

gene	HC Mean	UC Mean	logFC	pValue	fdr
IFNA21	3.695085	3.693392	-0.00066	0.982254	0.982254
ABCA1	5.452483	5.453955	0.000389	0.967391	0.96888
SEC24A	5.960605	5.944725	-0.00385	0.958766	0.961721
B4GALT4	6.943117	6.93343	-0.00201	0.950625	0.955026
CYP2E1	4.100775	4.100056	-0.00025	0.945839	0.951686
VAC14	4.816644	4.805554	-0.00333	0.933403	0.940627
SLC44A3	7.659109	7.634359	-0.00467	0.932925	0.940627
NSMAF	5.50856	5.513651	0.001332	0.917166	0.927135
MAPK13	7.92605	7.922087	-0.00072	0.905725	0.916993
PLD3	5.635841	5.637757	0.00049	0.89573	0.908287
ACHE	4.808469	4.827541	0.005711	0.893828	0.907772
FCER1A	5.297376	5.260303	-0.01013	0.889075	0.904356
IL6	4.292786	4.359378	0.022208	0.886226	0.902869
PPP2CB	6.742781	6.726546	-0.00348	0.881006	0.898957
MTMR1	5.121403	5.117775	-0.00102	0.878635	0.897945
CASP7	7.509155	7.517152	0.001536	0.874843	0.895476
PSAP	8.252609	8.256614	0.0007	0.863485	0.885242
ALDH7A1	6.360823	6.344009	-0.00382	0.862541	0.885242
NFYB	5.142265	5.143773	0.000423	0.860651	0.885125
DGKQ	5.586348	5.578951	-0.00191	0.854515	0.880205
PLA2G15	4.739257	4.729393	-0.00301	0.844151	0.870907
SMPD1	5.790136	5.790879	0.000185	0.84274	0.870831
PCCA	6.237958	6.253507	0.003592	0.84227	0.870831
VAPA	5.782859	5.778568	-0.00107	0.841329	0.870831
MED8	6.102134	6.11158	0.002231	0.841329	0.870831
RXRG	3.824935	3.839925	0.005643	0.838509	0.870831
ASAH1	6.108317	6.113085	0.001126	0.831937	0.866546
TNFRSF10I	5.340955	5.341434	0.000129	0.830062	0.865978
CHUK	6.206633	6.21639	0.002266	0.826782	0.863941
INPP5K	5.168225	5.162646	-0.00156	0.823038	0.861411
ROCK1	5.224451	5.231861	0.002045	0.821634	0.861327
MED7	6.116453	6.115513	-0.00022	0.821634	0.861327
EBP	6.939079	6.943018	0.000819	0.816961	0.859195
LIPF	3.700695	3.688068	-0.00493	0.813694	0.857144
B3GALNT1	4.055306	4.060729	0.001928	0.813694	0.857144
GNAI1	5.532774	5.520455	-0.00322	0.810896	0.85697
PIK3C2A	4.719126	4.73426	0.004619	0.809032	0.85639
FAAH2	5.562335	5.568222	0.001526	0.8081	0.85639
OSBPL7	5.232349	5.228661	-0.00102	0.807635	0.85639
FIG4	6.252158	6.253075	0.000212	0.806238	0.85639
PPP2R5A	6.780632	6.786927	0.001339	0.806238	0.85639
B3GNT3	7.343568	7.370995	0.005378	0.800657	0.854472
NCOR1	5.763345	5.75634	-0.00175	0.799264	0.854385
SUMF1	6.681926	6.701096	0.004133	0.78213	0.837445
IKBKB	5.297032	5.292658	-0.00119	0.779823	0.83635
ANKRD1	3.354127	3.351784	-0.00101	0.771992	0.829319
MED22	5.188373	5.195234	0.001906	0.771073	0.829319
B3GNT2	5.496058	5.516077	0.005245	0.768775	0.828597
CYP1B1	3.856326	3.860262	0.001472	0.767397	0.828483
DDIT3	5.066725	5.05878	-0.00226	0.758688	0.820442
G0S2	5.208433	5.205898	-0.0007	0.755944	0.818835
PAFAH1B3	5.83518	5.840594	0.001338	0.747276	0.810794
ACOT12	3.300069	3.264226	-0.01575	0.738184	0.802267
HMGCR	7.157689	7.151711	-0.00121	0.736825	0.802129
MBTPS1	5.978718	5.965643	-0.00316	0.729131	0.795083
S1PR3	4.085771	4.137079	0.018004	0.729131	0.795083
MAPK10	4.616935	4.636874	0.006217	0.727325	0.795083

CALM2	7.880967	7.874499	-0.00118	0.726419	0.795083
PSAPL1	4.007024	4.002854	-0.0015	0.724619	0.795083
MAP2K2	6.076993	6.082498	0.001306	0.723267	0.795083
PRKCE	4.191651	4.18292	-0.00301	0.719666	0.792728
ACAT2	6.580192	6.606009	0.005649	0.717418	0.791591
SMARCD3	4.544621	4.556992	0.003922	0.707558	0.782038
PNPLA7	5.113264	5.110353	-0.00082	0.702646	0.77793
ELOVL1	6.951571	6.943506	-0.00167	0.701309	0.777772
NFATC3	5.136448	5.126381	-0.00283	0.700863	0.777772
CALML4	7.497095	7.515601	0.003557	0.699081	0.777772
EHHADH	5.933484	5.892468	-0.01001	0.693304	0.772844
MED19	6.155097	6.149596	-0.00129	0.6902	0.770703
APAF1	4.81223	4.809349	-0.00086	0.676517	0.756723
CALML5	4.279573	4.284346	0.001608	0.671687	0.752614
PLA2G2D	4.652603	4.721913	0.021333	0.670372	0.752435
DBI	8.718369	8.737222	0.003116	0.665557	0.748321
PGS1	4.748657	4.750634	0.000601	0.664249	0.748143
PTGIS	4.125902	4.140528	0.005105	0.659455	0.744029
ROCK2	6.646181	6.642348	-0.00083	0.657279	0.742862
MED26	4.966841	4.949418	-0.00507	0.654239	0.740712
FAR2	6.549847	6.595541	0.01003	0.649041	0.736107
PPP2R1B	5.02414	5.018315	-0.00167	0.643429	0.731016
GC	3.421399	3.424436	0.00128	0.642136	0.730823
GALC	5.588645	5.577182	-0.00296	0.63141	0.719874
ACOT7	5.626605	5.645491	0.004834	0.630555	0.719874
HSPA8	7.978828	7.977185	-0.0003	0.630555	0.719874
SEC24C	6.374373	6.377174	0.000634	0.630127	0.719874
CREBBP	5.578681	5.561651	-0.00441	0.625436	0.718094
SLC44A2	6.882613	6.895129	0.002621	0.622884	0.716426
MAPK1	5.331157	5.32866	-0.00068	0.616523	0.710365
NFYC	5.822237	5.813738	-0.00211	0.612719	0.707235
ARSB	4.228413	4.238381	0.003397	0.608086	0.703133
PLA2G4F	5.472366	5.481815	0.002489	0.601791	0.697093
AGER	4.508276	4.521303	0.004163	0.597195	0.693001
RAB5A	6.461093	6.487449	0.005873	0.597194	0.693001
PPP1CC	8.140089	8.12139	-0.00332	0.596359	0.693001
DHCR7	5.281064	5.302358	0.005805	0.593862	0.692839
CSNK2A1	5.613984	5.603232	-0.00277	0.592614	0.692624
CAV1	5.984406	6.021822	0.008992	0.587635	0.68804
B3GNT5	5.971845	5.951408	-0.00495	0.586807	0.68804
PLEKHA1	6.251883	6.229618	-0.00515	0.585566	0.68804
ABCG1	6.026098	5.993522	-0.00782	0.582676	0.685935
ALDH3B1	5.208992	5.183899	-0.00697	0.582263	0.685935
ADORA1	4.363155	4.370894	0.002557	0.581851	0.685935
ORMDL3	5.759302	5.735797	-0.0059	0.579792	0.685935
POU2F1	4.883564	4.888083	0.001334	0.576504	0.683614
FADS2	3.942403	3.968574	0.009545	0.573226	0.680967
EIF2AK3	5.937119	5.962106	0.006059	0.571589	0.680264
ACAA2	7.971082	7.942993	-0.00509	0.571588	0.680264
ACSM3	5.652264	5.620492	-0.00813	0.565067	0.67497
B4GALT2	4.597725	4.601709	0.00125	0.553338	0.662175
PPP1CB	6.996144	7.021056	0.005128	0.552533	0.662175
ELOVL7	5.60502	5.611755	0.001732	0.54892	0.659312
NOS3	4.517262	4.551549	0.010909	0.546918	0.658121
PRKCA	5.25927	5.22051	-0.01067	0.539343	0.650208
FAS	6.128778	6.148665	0.004674	0.538151	0.649975
HRAS	5.64235	5.675617	0.008481	0.536962	0.649744
VAV3	6.508724	6.466256	-0.00944	0.535773	0.649512

MVK	4.629366	4.618707	-0.00333	0.531032	0.644966
SP1	5.65903	5.672636	0.003465	0.530637	0.644966
MAPK11	4.207348	4.220945	0.004655	0.530244	0.644966
UGCG	6.70397	6.708188	0.000908	0.525135	0.641394
ACADVL	7.412707	7.385733	-0.00526	0.52396	0.641161
DGKZ	4.852929	4.864763	0.003514	0.519662	0.6371
FAR1	5.86018	5.847455	-0.00314	0.519662	0.6371
SELE	4.608958	4.787253	0.054757	0.507264	0.624251
HSP90AA1	8.106811	8.101039	-0.00103	0.503037	0.620221
GNA13	6.218905	6.199697	-0.00446	0.498831	0.616203
HSP90AB1	7.48268	7.460687	-0.00425	0.496162	0.614071
MTMR12	5.278896	5.270518	-0.00229	0.49123	0.609125
PIP5K1C	6.091836	6.105562	0.003247	0.490852	0.609125
ALDH2	8.93839	8.963539	0.004053	0.488583	0.60816
AGK	4.822333	4.834334	0.003586	0.48557	0.605568
BCL2L1	5.01141	5.024683	0.003816	0.484067	0.604852
TNFRSF1A	6.998244	7.007805	0.00197	0.480694	0.601791
CYP11A1	4.059498	4.051643	-0.00279	0.4751	0.595935
AACS	5.636471	5.620993	-0.00397	0.473615	0.595219
MIB2	5.272112	5.255217	-0.00463	0.473244	0.595219
ALDH1B1	5.688255	5.669086	-0.00487	0.470652	0.593788
PIK3C2B	7.63344	7.617228	-0.00307	0.464021	0.586559
MAPK9	5.597844	5.606701	0.002281	0.462556	0.585844
ECHS1	8.450798	8.414275	-0.00625	0.461823	0.585844
AKR1C4	4.68971	4.704837	0.004646	0.458902	0.583486
PCTP	6.379113	6.362367	-0.00379	0.457445	0.582772
ENPP7	4.617589	4.575656	-0.01316	0.455627	0.581594
HSD17B7	6.726096	6.758639	0.006963	0.455264	0.581594
PIP5K1B	7.158491	7.171709	0.002661	0.448396	0.574618
PON2	7.213377	7.193051	-0.00407	0.448396	0.574618
ICAM1	4.868492	4.944431	0.02233	0.439451	0.565381
OLR1	3.618422	3.571875	-0.01868	0.439095	0.565381
MED28	6.096166	6.087081	-0.00215	0.439095	0.565381
PLCG1	5.276042	5.292691	0.004545	0.43874	0.565381
ENPP2	6.799474	6.842229	0.009043	0.436256	0.565381
GPS2	6.445079	6.412564	-0.0073	0.43484	0.565032
FUT2	7.136628	7.175933	0.007924	0.432017	0.562486
PNPLA6	5.974148	5.922848	-0.01244	0.428853	0.559486
ESYT1	6.460897	6.479428	0.004132	0.428151	0.559486
PI4K2B	6.001192	6.026249	0.006011	0.426402	0.558526
PLA2G6	4.542926	4.521217	-0.00691	0.422218	0.554162
ERN1	4.557416	4.539148	-0.00579	0.420829	0.553455
PIK3CA	4.906681	4.899184	-0.00221	0.419443	0.552748
AGPAT2	6.953308	6.91756	-0.00744	0.419097	0.552748
ABHD5	5.56573	5.544171	-0.0056	0.419097	0.552748
PI4KA	5.500531	5.470141	-0.00799	0.416679	0.55246
PIAS4	5.534802	5.548993	0.003694	0.416333	0.55246
BAX	6.20897	6.225107	0.003745	0.412552	0.549225
PPARD	5.214104	5.203368	-0.00297	0.410497	0.54761
PRKACB	6.349317	6.33144	-0.00407	0.408449	0.545997
SPTLC2	5.95232	5.994449	0.010175	0.406747	0.54484
RUFY1	5.432099	5.411297	-0.00554	0.403017	0.540957
PLIN2	6.037419	5.952443	-0.02045	0.399981	0.537991
HDAC3	6.27195	6.293375	0.00492	0.399644	0.537991
HSD17B4	6.968822	6.980224	0.002359	0.398634	0.537991
NR1H4	5.382439	5.235872	-0.03983	0.39562	0.535444
MED11	6.388394	6.354733	-0.00762	0.393616	0.533842
CLOCK	4.758243	4.77087	0.003823	0.389297	0.529087

PPARG	7.423978	7.366601	-0.01119	0.386983	0.527042
MAP3K7	5.830854	5.824396	-0.0016	0.384021	0.524104
PI4KB	5.884	5.857096	-0.00661	0.383364	0.524104
DHRS7B	6.460532	6.424403	-0.00809	0.381399	0.522717
HSD17B12	6.383413	6.410546	0.006119	0.380745	0.522717
LBR	6.480604	6.449604	-0.00692	0.379114	0.521783
MGLL	7.306872	7.25695	-0.00989	0.376189	0.518854
GDPD5	4.612065	4.618631	0.002052	0.371344	0.513258
CTSD	6.954184	6.919733	-0.00716	0.368136	0.509908
MED12	5.618539	5.598442	-0.00517	0.367497	0.509908
PEMT	5.283752	5.257024	-0.00732	0.36431	0.506764
LPCAT2	4.992385	5.040614	0.01387	0.363992	0.506764
PRKCB	4.670569	4.719411	0.015009	0.363357	0.506764
PPT1	6.371156	6.405937	0.007855	0.362722	0.506764
FABP5	7.450185	7.422874	-0.0053	0.361456	0.506764
CHD9	5.789876	5.770866	-0.00474	0.360823	0.506764
PPP1CA	7.523181	7.543489	0.003889	0.359874	0.506764
MORC2	4.87136	4.836647	-0.01032	0.356104	0.502871
SYNJ1	4.79468	4.772302	-0.00675	0.354227	0.501308
ACOT4	5.537567	5.48358	-0.01413	0.351113	0.497983
PTDSS2	5.490243	5.509857	0.005145	0.351113	0.497983
SGPP2	5.684431	5.728087	0.011038	0.349252	0.497512
IRAK1	6.233923	6.254278	0.004703	0.348633	0.497512
A4GALT	4.179619	4.203571	0.008244	0.348014	0.497512
SPTLC1	6.454169	6.429318	-0.00557	0.344015	0.493291
PIK3CG	4.194005	4.228361	0.01177	0.343709	0.493291
ALOX15B	4.088967	4.123319	0.01207	0.341874	0.492389
DEGS1	5.709861	5.738656	0.007257	0.339741	0.490402
SBF1	5.140511	5.152146	0.003262	0.336708	0.487105
MAP2K7	4.819593	4.799949	-0.00589	0.3355	0.486438
SIN3B	4.739013	4.754771	0.004789	0.333093	0.484025
THRAP3	5.436526	5.463658	0.007182	0.331893	0.483361
CIDEA	4.053043	4.049226	-0.00136	0.3298	0.48139
MAP2K3	6.951424	6.988989	0.007775	0.3298	0.48139
TIRAP	4.719461	4.736158	0.005095	0.328906	0.48139
PPP2R2C	3.92223	3.916639	-0.00206	0.32564	0.478537
ALOX5	5.5013	5.528108	0.007013	0.324754	0.478314
MBOAT2	6.045134	6.094674	0.011775	0.324458	0.478314
PPP2R1A	6.95486	6.930153	-0.00513	0.322102	0.476564
MCEE	6.484798	6.453792	-0.00691	0.315971	0.468558
CDS1	7.387118	7.32814	-0.01156	0.313655	0.466185
MAPK3	7.459702	7.409975	-0.00965	0.311351	0.46382
GGPS1	6.395772	6.368844	-0.00609	0.310203	0.46317
PMVK	6.49919	6.454137	-0.01004	0.309917	0.46317
HSPA2	5.705785	5.670718	-0.00889	0.309344	0.46317
SUMO2	7.457221	7.481663	0.004721	0.30877	0.46317
LDLR	5.833856	5.884576	0.012488	0.307631	0.46317
GPD2	6.093854	6.13312	0.009266	0.306208	0.462508
RELA	5.407498	5.4208	0.003545	0.304789	0.461436
BDKRB2	6.911836	6.951649	0.008286	0.302528	0.45908
ACOX3	5.74822	5.781781	0.008399	0.300278	0.456731
TRADD	5.295319	5.31008	0.004016	0.299998	0.456731
SLC25A17	5.16718	5.158445	-0.00244	0.298598	0.456309
ATF4	7.712929	7.735361	0.00419	0.29831	0.456309
DPEP2	5.537149	5.574705	0.009752	0.296925	0.455892
SREBF1	5.077939	5.04472	-0.00947	0.296646	0.455892
CAMK2B	3.899864	3.892385	-0.00277	0.296368	0.455892
ACER3	5.621105	5.659185	0.00974	0.290019	0.448461

PRKAG2	5.832677	5.810446	-0.00551	0.285385	0.442346
LPIN2	5.966932	5.928582	-0.0093	0.283761	0.44088
STARD4	5.204638	5.274456	0.019224	0.283492	0.44088
PIK3C3	5.30766	5.281311	-0.00718	0.281606	0.43963
GBA3	7.07786	6.796154	-0.05859	0.277862	0.434828
PPP2R5E	6.195526	6.168939	-0.0062	0.277596	0.434828
SLC27A3	5.680703	5.725049	0.011218	0.276534	0.434828
ELOVL3	3.920317	3.904554	-0.00581	0.275473	0.434221
ACSL5	7.992069	7.928808	-0.01147	0.273624	0.432353
FCER1G	7.131989	7.212681	0.016231	0.273098	0.432353
MTMR14	5.359411	5.336402	-0.00621	0.270476	0.429463
ALOX15	3.899585	3.946731	0.017338	0.268911	0.428022
POU2F3	4.015383	3.97548	-0.01441	0.268391	0.428022
SAMD8	4.740697	4.776172	0.010756	0.266316	0.425975
INPPL1	5.464128	5.428316	-0.00949	0.263482	0.42248
MED16	5.674676	5.710075	0.008972	0.259143	0.416548
AKT3	4.438162	4.478307	0.012991	0.258889	0.416548
KDSR	5.442054	5.402557	-0.01051	0.258636	0.416548
PTGS2	4.463559	4.534723	0.02282	0.251607	0.407454
PIK3R4	4.822052	4.796149	-0.00777	0.247651	0.402047
CD40LG	4.261041	4.305923	0.015116	0.247159	0.402047
PLD6	5.146471	5.116112	-0.00854	0.246669	0.402047
GLYCTK	5.954224	5.885828	-0.01667	0.24569	0.401869
ACP6	5.635422	5.596797	-0.00992	0.245201	0.401869
NCOA3	5.240322	5.28256	0.011582	0.244957	0.401869
PIK3C2G	3.55754	3.545441	-0.00491	0.24301	0.400505
ANGPTL4	4.744237	4.762773	0.005626	0.242283	0.400321
PLD4	3.955397	3.967492	0.004405	0.242283	0.400321
HMGCS2	8.174133	7.941759	-0.04161	0.242282	0.400321
SQLE	5.352228	5.404388	0.013991	0.240352	0.400176
MIB1	4.925211	4.952614	0.008004	0.237952	0.397197
DGKH	4.298878	4.276361	-0.00758	0.237474	0.397197
STARD3	5.106632	5.072373	-0.00971	0.236521	0.396843
CEPT1	6.18742	6.219337	0.007423	0.23652	0.396843
PPP2R2B	3.943526	3.91483	-0.01054	0.232969	0.392909
STS	4.874634	4.97055	0.028112	0.232498	0.392909
CEL	4.105422	4.079332	-0.0092	0.227597	0.385848
PIK3CB	5.601125	5.557953	-0.01116	0.226671	0.385282
PLCB1	3.870485	3.914439	0.016291	0.222997	0.380029
EP300	6.221129	6.184137	-0.0086	0.222541	0.380029
BID	5.560629	5.610238	0.012814	0.222086	0.380029
FASN	5.163525	5.224079	0.016821	0.221403	0.380029
RAP1B	6.859121	6.906926	0.01002	0.221403	0.380029
CPNE1	5.754642	5.753165	-0.00037	0.220723	0.380029
NSDHL	6.250161	6.288835	0.0089	0.219818	0.380029
KRAS	6.087109	6.074678	-0.00295	0.219818	0.380029
B4GALT6	4.139785	4.109502	-0.01059	0.218915	0.380029
PEX11A	6.204934	6.09584	-0.02559	0.215109	0.375432
TRAF3	5.632246	5.649129	0.004318	0.214665	0.375432
GLB1L	4.678974	4.623555	-0.01719	0.213556	0.374731
PDPK1	6.04003	6.005939	-0.00817	0.212672	0.374188
MED13	5.398652	5.359415	-0.01052	0.212452	0.374188
CERK	5.967277	5.914868	-0.01273	0.211571	0.374188
ST3GAL4	5.333238	5.505274	0.045803	0.210913	0.374126
MTMR2	5.730997	5.694181	-0.0093	0.210474	0.374126
ENPP6	3.730581	3.689566	-0.01595	0.205481	0.366488
RGL1	5.938657	5.975843	0.009005	0.202484	0.362135
PNLIPRP2	5.488999	5.256989	-0.06231	0.20121	0.360847

NAGA	5.427981	5.387922	-0.01069	0.199941	0.359562
PIP4K2B	4.697032	4.708234	0.003436	0.199519	0.359562
MTMR8	3.818103	3.800129	-0.00681	0.199309	0.359562
MBOAT7	5.644385	5.60414	-0.01032	0.198678	0.359562
TNFAIP8L1	5.25048	5.275256	0.006792	0.198468	0.359562
PTGES3	7.063796	7.026128	-0.00771	0.198468	0.359562
GM2A	5.171962	5.238305	0.018389	0.198258	0.359562
PLA2G7	6.66736	6.806684	0.029836	0.19763	0.359562
LPL	4.370707	4.483229	0.036671	0.195962	0.359562
TLR6	4.177202	4.219538	0.014548	0.193273	0.356432
GDPD3	7.802396	7.899888	0.017915	0.192041	0.355166
MED29	5.718451	5.69143	-0.00683	0.190611	0.353526
ST6GALNA	4.74091	4.80891	0.020546	0.189392	0.352269
OXCT1	4.957254	4.992849	0.010322	0.187372	0.349511
ARNTL	5.154835	5.200371	0.012688	0.184373	0.344905
PITPNM1	5.68434	5.722055	0.009541	0.181409	0.340338
AGPAT5	5.594201	5.550641	-0.01128	0.180039	0.338743
CPT1A	6.127363	6.042058	-0.02023	0.180039	0.338743
SRC	5.583673	5.609878	0.006755	0.180038	0.338743
ABHD3	7.596849	7.570867	-0.00494	0.179843	0.338743
ABCB11	3.747434	3.669089	-0.03048	0.178287	0.338743
RAC1	7.081287	7.107875	0.005407	0.177127	0.338151
LY96	6.654167	6.754568	0.021605	0.176164	0.337303
GPD1L	7.725648	7.653237	-0.01359	0.172352	0.330976
GLIPR1	5.609714	5.674305	0.016516	0.168417	0.324377
MED13L	5.67466	5.644433	-0.00771	0.16712	0.322834
RHOA	7.328564	7.373191	0.008759	0.166933	0.322834
MED30	5.449691	5.415819	-0.00899	0.166751	0.322834
AKR1B10	9.1624	9.032506	-0.0206	0.166013	0.322834
ST3GAL2	4.836863	4.871645	0.010337	0.165647	0.322834
MED4	5.996766	5.954171	-0.01028	0.164915	0.322834
PRKAB2	5.26313	5.186149	-0.02126	0.164549	0.322834
HEXB	8.292819	8.262179	-0.00534	0.160209	0.316048
MTF1	5.850671	5.897108	0.011406	0.158429	0.313488
CCL5	6.753892	6.639141	-0.02472	0.157368	0.312337
ITPR1	5.007068	5.073981	0.019152	0.156487	0.311539
PLA2G2A	9.05949	9.19599	0.021575	0.155958	0.311438
ST8SIA1	4.249775	4.329581	0.026841	0.153694	0.307861
MTMR6	5.771497	5.828127	0.014087	0.152311	0.306033
SCD	6.125588	6.195404	0.01635	0.146542	0.295352
ARSJ	4.475244	4.549999	0.0239	0.144546	0.292235
HSPA4	5.869431	5.840551	-0.00712	0.143721	0.291471
MED23	5.191641	5.172535	-0.00532	0.142571	0.290043
NCOA1	6.378948	6.350097	-0.00654	0.13997	0.285644
ACACB	5.564529	5.543153	-0.00555	0.138363	0.283253
RXRB	4.946787	4.924197	-0.0066	0.138043	0.283253
STARD3NL	6.1125	6.07981	-0.00774	0.132231	0.272413
MED31	5.700007	5.748896	0.012321	0.131923	0.272413
CD36	5.759955	5.634336	-0.03181	0.130694	0.270961
AMACR	5.559713	5.455338	-0.02734	0.128413	0.267083
CDS2	5.645793	5.68372	0.009659	0.128111	0.267083
PPP2R5C	6.017517	6.046606	0.006957	0.125866	0.263468
PNPLA2	5.077046	5.017987	-0.01688	0.125272	0.263072
PPP3R1	5.991333	6.050048	0.01407	0.124681	0.262677
TBL1X	4.755177	4.743735	-0.00348	0.121902	0.257656
MTMR9	5.400113	5.377019	-0.00618	0.121756	0.257656
POU2F2	4.478059	4.550577	0.023176	0.121461	0.257656
CGA	3.290893	3.276844	-0.00617	0.120458	0.257108

CDC42	6.265537	6.304649	0.008978	0.119598	0.256114
CSNK1G2	5.994853	6.031229	0.008728	0.119455	0.256114
PLA2G4D	4.551684	4.599932	0.015212	0.115095	0.248103
ORMDL2	6.851534	6.906986	0.011629	0.114818	0.248103
PLA2G12B	5.183049	5.002908	-0.05103	0.112894	0.244981
PTGES	4.822497	4.870996	0.014436	0.110996	0.241666
ACOT11	6.102215	6.028546	-0.01752	0.110995	0.241666
HSD17B2	8.870569	8.711877	-0.02604	0.110726	0.241666
PLA2R1	3.907587	3.867676	-0.01481	0.109255	0.240288
MED25	4.893661	4.934186	0.011898	0.108592	0.239638
STARD10	6.937691	6.869651	-0.01422	0.108195	0.239575
MYD88	6.577159	6.623021	0.010025	0.107143	0.238055
NLRP3	4.523223	4.596059	0.023046	0.106099	0.236543
GPX2	8.942133	9.066427	0.019915	0.104546	0.233881
SPHK1	3.873241	3.97479	0.037338	0.104419	0.233881
PLEKHA4	4.68499	4.75552	0.021557	0.104292	0.233881
MOGAT2	5.85391	5.744799	-0.02714	0.104035	0.233881
HSPD1	6.010943	5.983922	-0.0065	0.102256	0.231946
NCOR2	5.256582	5.284172	0.007552	0.101626	0.231325
MS4A2	4.893934	4.959036	0.019065	0.098647	0.225331
PAFAH1B2	5.141993	5.166343	0.006816	0.097063	0.222493
AKR1D1	4.987327	4.955527	-0.00923	0.094427	0.217214
ARSA	5.311721	5.375522	0.017226	0.094191	0.217214
MTMR3	5.568348	5.530972	-0.00972	0.093837	0.217214
ARF3	6.752468	6.716647	-0.00767	0.092898	0.215988
ACSL4	4.615466	4.728036	0.034765	0.091504	0.213509
CCNC	5.892105	5.933992	0.01022	0.091388	0.213509
AGPAT3	5.389546	5.349441	-0.01078	0.091273	0.213509
NCF2	5.82315	5.983747	0.039249	0.089784	0.211774
KPNB1	7.312239	7.346008	0.006647	0.08899	0.210664
NDUFAB1	7.916321	7.870169	-0.00844	0.088877	0.210664
GDE1	6.75973	6.705758	-0.01157	0.08854	0.210664
INPP4B	4.966471	5.02613	0.017227	0.086866	0.207903
PAFAH2	5.926571	5.851452	-0.0184	0.086755	0.207903
POMC	4.146907	4.209901	0.021751	0.086313	0.207903
ACSL6	3.646775	3.674091	0.010766	0.085436	0.206761
SLC25A20	6.708776	6.621786	-0.01883	0.085217	0.206761
TRAF6	5.182591	5.136141	-0.01299	0.085217	0.206761
THEM4	4.764766	4.730821	-0.01031	0.085217	0.206761
GPX1	7.325963	7.378162	0.010243	0.083809	0.205885
PTGR1	6.605468	6.445388	-0.03539	0.083379	0.205606
DGKA	6.038447	5.991172	-0.01134	0.081364	0.201399
NOX1	6.617258	6.774306	0.033839	0.080527	0.200087
SLC27A1	4.548685	4.579095	0.009613	0.080319	0.200087
PLD2	4.73233	4.761396	0.008834	0.080111	0.200087
SEC24D	5.120257	5.200398	0.022406	0.078364	0.196968
SLCO1B1	3.708275	3.6836	-0.00963	0.07816	0.196968
PRKD2	5.816698	5.879876	0.015585	0.077957	0.196968
CCL2	6.16726	6.325445	0.036537	0.077451	0.196955
SPTLC3	4.539844	4.4739	-0.02111	0.077451	0.196955
SEC23A	5.821834	5.740365	-0.02033	0.076847	0.196955
MBOAT1	6.443386	6.382655	-0.01366	0.075751	0.194916
MTMR4	6.342642	6.284016	-0.0134	0.075652	0.194916
BAD	5.494207	5.437245	-0.01504	0.074961	0.194421
CYP2C19	4.064723	3.986215	-0.02814	0.074569	0.194178
LYPLA1	5.791177	5.706667	-0.02121	0.074276	0.194178
CYP39A1	4.593559	4.670035	0.023821	0.073887	0.193953
ELOVL5	4.654147	4.724933	0.021777	0.073693	0.193953

TBK1	5.822013	5.852361	0.007501	0.073692	0.193953
MTMR10	5.687612	5.659683	-0.0071	0.072729	0.19325
PPP2R2A	6.22737	6.266244	0.008978	0.071586	0.190993
SLC10A2	4.385752	4.009198	-0.12951	0.067611	0.181129
AGT	5.42513	5.429485	0.001157	0.06761	0.181129
SMPD3	6.22938	6.11856	-0.0259	0.06725	0.181129
VAV1	5.289213	5.379261	0.024355	0.066981	0.181129
SLC25A1	6.643505	6.560629	-0.01811	0.066356	0.180744
DHCR24	6.577408	6.677014	0.021684	0.066267	0.180744
GNAI2	5.953947	6.038407	0.020322	0.06556	0.180082
GBGT1	4.543191	4.597274	0.017073	0.065121	0.179635
CYP2J2	8.217746	7.996084	-0.03945	0.06356	0.176075
MID1IP1	6.237202	6.311344	0.017048	0.063304	0.176075
TRIB3	4.239067	4.298235	0.019998	0.063303	0.176075
CBR4	6.367282	6.321519	-0.01041	0.063133	0.176075
LIPC	3.959974	3.829145	-0.04847	0.062708	0.176075
SUMF2	6.352692	6.29831	-0.0124	0.061112	0.172974
ACSL1	5.251234	5.398735	0.039965	0.060451	0.171849
SEC24B	6.099007	6.046318	-0.01252	0.059632	0.170265
SLC44A1	7.820611	7.75668	-0.01184	0.058661	0.168231
PPP2R5B	4.784744	4.846802	0.018592	0.056527	0.162828
MAPK14	5.369989	5.34085	-0.00785	0.056449	0.162828
OSBPL5	5.843448	5.906885	0.015578	0.055984	0.162704
MTM1	6.390604	6.313398	-0.01754	0.055599	0.16231
HSPA5	6.901184	6.985063	0.017429	0.054912	0.161024
FYN	5.401181	5.48934	0.023358	0.054836	0.161024
PPP3CA	6.325322	6.370207	0.010201	0.05476	0.161024
PIP5K1A	4.6697	4.628221	-0.01287	0.054608	0.161024
PLCB4	5.600699	5.686256	0.021872	0.052232	0.155977
NFATC1	4.223209	4.294627	0.024193	0.051579	0.154738
TXNRD1	5.521979	5.607566	0.022189	0.051579	0.154738
NFYA	4.702512	4.678143	-0.0075	0.05072	0.153575
HSD3B7	5.12832	5.17568	0.013262	0.050224	0.152784
BMX	4.902487	4.808145	-0.02803	0.049384	0.150933
ALPI	5.088111	4.936782	-0.04356	0.046336	0.142287
DGKE	4.380989	4.411174	0.009906	0.045421	0.140138
FDPS	6.635058	6.69127	0.012171	0.045162	0.140002
PECR	5.357404	5.291264	-0.01792	0.043825	0.136507
OSBPL1A	5.511758	5.408176	-0.02737	0.0432	0.135206
INPP4A	5.167717	5.225438	0.016025	0.0432	0.135206
ESRRA	6.443858	6.364592	-0.01786	0.043075	0.135206
LTA4H	7.501914	7.437347	-0.01247	0.04185	0.132901
RAB14	5.677653	5.614893	-0.01604	0.041489	0.132399
RAC2	6.70828	6.853767	0.030954	0.041309	0.132399
SACM1L	6.603478	6.556648	-0.01027	0.040952	0.131978
OSBPL3	5.32916	5.443699	0.030679	0.039605	0.128273
ACADS	6.654813	6.489024	-0.0364	0.03949	0.128273
MED10	6.123866	6.163776	0.009372	0.03796	0.124182
CSNK2B	6.566536	6.533669	-0.00724	0.036914	0.12137
CRLS1	6.7543	6.674681	-0.01711	0.036536	0.120735
AGPAT1	5.489416	5.424477	-0.01717	0.03469	0.115222
GSTM4	5.852287	5.725376	-0.03163	0.034127	0.113932
GLB1	7.141072	7.191025	0.010057	0.033722	0.113161
PLD1	6.366043	6.289311	-0.01749	0.033421	0.112733
SYNJ2	4.616725	4.573874	-0.01345	0.033123	0.112308
HSD3B1	4.224843	4.001943	-0.0782	0.032876	0.112054
IKBKG	4.971398	5.033331	0.017862	0.03263	0.111802
PIP4K2A	5.378134	5.45481	0.020423	0.032533	0.111802

PPP2R5D	5.225	5.160844	-0.01782	0.032435	0.111802
MED18	3.620187	3.589902	-0.01212	0.032435	0.111802
CROT	5.377959	5.244773	-0.03618	0.03176	0.111159
NFE2L2	6.394073	6.34597	-0.01089	0.031759	0.111159
RAB4A	6.064593	5.993178	-0.01709	0.031237	0.110519
ABCB4	4.596365	4.512123	-0.02669	0.030816	0.109623
IRAK4	5.490012	5.440721	-0.01301	0.030583	0.109394
ARSD	5.65856	5.561021	-0.02509	0.030261	0.108838
HMGCL	6.703971	6.607452	-0.02092	0.030078	0.108781
BDH1	5.923191	5.819659	-0.02544	0.029987	0.108781
HSPA1L	4.216106	4.175975	-0.0138	0.029266	0.107034
BDH2	5.504561	5.398554	-0.02805	0.029221	0.107034
THRSP	3.717006	3.692264	-0.00964	0.028691	0.106125
GRHL1	4.088723	4.197003	0.037709	0.02843	0.105758
PLEKHA5	4.931594	4.853349	-0.02307	0.028126	0.105232
INPP5E	4.837173	4.792782	-0.0133	0.028083	0.105232
MED20	5.786538	5.721395	-0.01633	0.027912	0.105232
SCAP	6.768549	6.681825	-0.0186	0.027571	0.104964
ACOT9	5.982238	6.06569	0.019987	0.027318	0.104613
ALDH9A1	7.27407	7.220458	-0.01067	0.02715	0.104583
HSD11B2	9.40086	9.243848	-0.0243	0.026652	0.103277
TSPO	7.806242	7.893831	0.016097	0.026571	0.103277
ST3GAL1	4.727165	4.826604	0.030033	0.025882	0.1015
OSBPL6	4.106451	4.019731	-0.03079	0.025404	0.100231
MLYCD	5.809047	5.713867	-0.02383	0.025286	0.100231
CRAT	6.102013	5.968103	-0.03201	0.025169	0.100231
FADS1	4.419872	4.535185	0.037157	0.024358	0.097883
CYP3A4	5.763705	5.488922	-0.07047	0.024244	0.097883
CPT2	7.014032	6.886492	-0.02647	0.023981	0.097571
CYP4F2	4.942551	4.735677	-0.06168	0.023497	0.096205
SGMS1	5.005786	5.133792	0.036428	0.022986	0.094707
RAP1A	5.55353	5.503458	-0.01307	0.022986	0.094707
MAP2K1	6.427008	6.522906	0.021368	0.02277	0.094707
BCL2	4.995301	5.058851	0.018238	0.02175	0.091348
PIK3R2	5.779582	5.697969	-0.02052	0.021646	0.091348
IRF7	6.176106	6.266468	0.020955	0.021509	0.091348
ACBD5	5.738628	5.655712	-0.021	0.02117	0.09067
FUT3	8.111437	8.244179	0.023418	0.021102	0.09067
IL18	7.972491	8.113758	0.02534	0.020969	0.09067
PPARGC1E	5.220015	5.127951	-0.02567	0.02077	0.09067
PPP2R3A	5.124257	4.997487	-0.03614	0.02067	0.09067
PTEN	6.60637	6.564541	-0.00916	0.020213	0.089515
VCAM1	6.682756	6.834747	0.032445	0.019797	0.088271
PLCB2	4.427174	4.494529	0.021784	0.019701	0.088271
CTSA	7.983268	7.856393	-0.02311	0.019419	0.087788
PPP3CC	5.993102	5.908866	-0.02042	0.019232	0.087553
MVD	5.128161	5.217368	0.024881	0.018986	0.08704
VLDLR	4.444186	4.267181	-0.05864	0.018864	0.08704
ACAA1	7.320603	7.22802	-0.01836	0.018712	0.08701
HSD3B2	4.44991	4.215815	-0.07796	0.018591	0.08701
CYBB	5.186457	5.298091	0.030723	0.018293	0.086294
PON3	5.492516	5.329781	-0.04339	0.017853	0.084833
XBP1	7.305673	7.449971	0.028218	0.017479	0.083667
PLEKHA6	6.716099	6.60361	-0.02437	0.017422	0.083667
CH25H	5.471378	5.604424	0.034662	0.017365	0.083667
ACADM	6.72963	6.597884	-0.02852	0.016945	0.082942
HSD17B11	8.327009	8.191668	-0.02364	0.016615	0.081943
INPP5J	5.602793	5.440679	-0.04236	0.016318	0.081092

RAF1	6.208069	6.147217	-0.01421	0.016318	0.081092
GLTP	7.794235	7.689063	-0.0196	0.015894	0.080208
OSBPL10	4.918432	4.992458	0.021552	0.015868	0.080208
VAV2	5.101307	5.019117	-0.02343	0.015816	0.080208
ACOX1	6.510243	6.377884	-0.02963	0.015737	0.080208
GPX4	7.181296	7.051216	-0.02637	0.014851	0.077344
SGMS2	5.692132	5.783959	0.023088	0.014826	0.077344
LPCAT3	6.433954	6.332623	-0.0229	0.014728	0.077344
SLC22A5	6.971823	6.791145	-0.03788	0.014704	0.077344
IKBKE	5.123082	5.192795	0.019499	0.01463	0.077344
CSNK2A2	5.959049	5.913856	-0.01098	0.014461	0.077344
ACAD10	5.022385	4.959291	-0.01824	0.013962	0.076381
ACSF3	4.518654	4.423853	-0.03059	0.013869	0.076381
PAFAH1B1	6.938707	6.8896	-0.01025	0.013799	0.076381
ST3GAL5	5.45568	5.557491	0.026675	0.013615	0.076381
MED14	5.23936	5.178521	-0.01685	0.013524	0.076381
PIK3CD	4.647601	4.720938	0.022587	0.013054	0.074547
TNF	5.213508	5.375194	0.044062	0.012556	0.072338
PPP2R2D	5.572236	5.483584	-0.02314	0.012472	0.072338
SCP2	6.535928	6.413049	-0.02738	0.012366	0.072338
CYP2B6	5.353053	5.188024	-0.04518	0.012158	0.071952
ELOVL4	4.246585	4.176552	-0.02399	0.012137	0.071952
PIK3R1	5.901062	5.811358	-0.0221	0.01177	0.070949
PLA2G12A	5.741024	5.656385	-0.02143	0.01165	0.070882
GCNT2	5.274665	5.110899	-0.0455	0.011433	0.070217
SPHK2	6.048273	5.942666	-0.02541	0.011394	0.070217
CDIPT	5.834994	5.770496	-0.01604	0.01084	0.067855
CDK8	5.877024	5.82	-0.01407	0.010508	0.066418
TECR	6.104509	6.031166	-0.01744	0.010274	0.065575
NUDT7	5.570947	5.466875	-0.02721	0.010045	0.064748
GAB2	5.448555	5.385453	-0.01681	0.009907	0.064492
PLEKHA3	5.630089	5.570136	-0.01545	0.009821	0.064492
NCF4	6.312988	6.445064	0.029872	0.009685	0.064334
FDFT1	7.409	7.494944	0.016639	0.009617	0.064334
ARV1	6.05563	5.965816	-0.02156	0.008888	0.06027
HSD17B8	6.527549	6.435356	-0.02052	0.00881	0.06027
PRKCZ	5.770078	5.671358	-0.0249	0.007781	0.053887
SLC33A1	6.24535	6.326389	0.0186	0.007781	0.053887
MMP1	6.077531	6.341453	0.061328	0.007657	0.053887
OCRL	5.649508	5.561608	-0.02262	0.007657	0.053887
AKR1B1	6.884908	6.754607	-0.02757	0.007575	0.053887
ALOX5AP	6.773351	6.937163	0.034476	0.007507	0.053887
PPT2	5.04211	4.979408	-0.01805	0.0074	0.053887
NR1H3	7.069687	6.929406	-0.02891	0.007321	0.053887
CYP27B1	4.122896	4.241588	0.040946	0.007088	0.053652
HADH	8.139002	8.008221	-0.02337	0.007088	0.053652
TANK	5.717147	5.817116	0.025009	0.006886	0.053365
GBA2	6.283888	6.19393	-0.0208	0.006762	0.05304
NUDT19	6.146992	6.07155	-0.01782	0.006738	0.05304
DECR2	6.18743	6.055946	-0.03099	0.006404	0.051467
AHR	6.123785	6.263221	0.032481	0.006369	0.051467
HSD17B3	4.615033	4.512613	-0.03238	0.006231	0.051346
VDR	7.348071	7.212871	-0.02679	0.005478	0.045724
LSS	4.949734	5.028731	0.022844	0.005468	0.045724
CYP27A1	6.554889	6.383303	-0.03827	0.005408	0.045724
PPARA	4.95528	4.866416	-0.02611	0.005378	0.045724
ACOT8	5.290691	5.17668	-0.03143	0.005348	0.045724
ABO	4.909231	4.768969	-0.04182	0.005086	0.04536

GNPAT	6.879611	6.828234	-0.01081	0.004974	0.044971
CHPT1	7.097998	6.930143	-0.03453	0.004873	0.044676
HADHA	6.782168	6.682265	-0.02141	0.004478	0.041642
IL1B	6.088773	6.528212	0.100536	0.004073	0.038428
TNFRSF10/	5.310293	5.40185	0.024662	0.004004	0.038331
MMAA	5.647672	5.735437	0.022247	0.003846	0.037374
HADHB	7.410628	7.313138	-0.01911	0.003774	0.037221
MED9	5.096138	5.000712	-0.02727	0.003766	0.037221
LGMN	6.97174	6.858342	-0.02366	0.003745	0.037221
CASP6	6.670995	6.541164	-0.02835	0.003604	0.037221
LPIN1	5.606157	5.72835	0.031107	0.003487	0.036619
GPAM	4.807743	4.717463	-0.02735	0.003394	0.036226
NCOA6	6.402454	6.347735	-0.01238	0.003304	0.035844
MAP3K5	6.222538	6.350514	0.02937	0.003272	0.035844
EPHX2	7.895076	7.629838	-0.0493	0.003196	0.035844
DECR1	8.490733	8.406094	-0.01445	0.002899	0.033107
MMP9	6.514807	6.950284	0.09335	0.002596	0.030178
PPARGC1/	5.582881	5.361633	-0.05834	0.002586	0.030178
PNPLA4	5.435979	5.289771	-0.03933	0.002461	0.029663
FAAH	6.268313	6.087462	-0.04224	0.002393	0.029392
VAPB	5.522596	5.447709	-0.0197	0.002383	0.029392
AGPAT4	4.716155	4.908398	0.057641	0.00236	0.029392
FHL2	7.059989	7.219987	0.03233	0.002267	0.029392
GDPD1	4.265862	4.181638	-0.02877	0.002258	0.029392
MED1	5.26033	5.18793	-0.01999	0.002231	0.029392
GAL3ST1	4.939414	4.779121	-0.04759	0.0022	0.029392
TBXAS1	5.48103	5.634635	0.039875	0.002109	0.029392
ATF6	5.754009	5.825431	0.017797	0.001826	0.026419
S1PR1	5.134821	5.356356	0.060938	0.001804	0.026419
CYCS	6.563364	6.461202	-0.02263	0.001432	0.021684
KNG1	3.97356	3.816128	-0.05832	0.001426	0.021684
ACOX2	6.241064	5.968187	-0.0645	0.001397	0.021684
CPNE3	6.713032	6.615418	-0.02113	0.001362	0.021684
GNAI3	6.09878	6.19682	0.023007	0.001315	0.021684
S1PR4	5.11407	5.300611	0.051687	0.001298	0.021684
LYN	6.467014	6.68175	0.047126	0.001296	0.021684
PHYH	6.997439	6.788322	-0.04377	0.001288	0.021684
CXCL3	5.737023	6.042084	0.074744	0.000886	0.016473
MED17	5.334362	5.263698	-0.01924	0.000857	0.016417
RXRA	6.446793	6.336873	-0.02481	0.000803	0.01585
PTPN13	4.221785	4.366864	0.048745	0.000746	0.01518
GNA12	4.842676	4.909966	0.019908	0.000691	0.014514
CYBA	6.357873	6.474216	0.026161	0.000687	0.014514
NEU1	6.525062	6.368361	-0.03507	0.000656	0.014514
CD40	5.394953	5.589331	0.051065	0.000623	0.014489
CHKA	5.716241	5.597734	-0.03022	0.000463	0.011164
ACSF2	6.506352	6.189522	-0.07202	0.000412	0.010305
PLA2G3	3.875883	4.135223	0.09344	0.000404	0.010305
HSP90B1	7.964975	8.107745	0.025631	0.000365	0.009892
CAMK2G	5.176317	5.060834	-0.03255	0.000348	0.009859
SRD5A3	4.8524	5.102003	0.072365	0.000321	0.009508
SPNS2	5.528845	5.777909	0.063569	0.000317	0.009508
STAT3	6.434815	6.579002	0.03197	0.000233	0.007597
PTGDS	6.669264	7.07445	0.08509	0.000221	0.007556
JAK2	5.051364	5.222496	0.048067	0.000212	0.007556
INPP5D	4.697074	4.846298	0.045121	0.000176	0.006735
ACAT1	6.351707	6.211955	-0.0321	0.00017	0.006735
LIPG	5.323109	5.58099	0.068252	0.000131	0.005668

TNFAIP8	5.921187	6.111925	0.04574	0.000125	0.005668
MMP3	5.53301	6.211386	0.166851	7.25E-05	0.003633
ME1	5.108004	5.391712	0.077984	4.33E-05	0.002348
FDX1	5.990237	5.820496	-0.04147	3.27E-05	0.001937
CASP1	7.388149	7.736518	0.066471	1.98E-05	0.001292
TLR4	5.156469	5.326064	0.046686	1.93E-05	0.001292
DGKD	6.719637	6.536516	-0.03986	1.89E-05	0.001292
PLA1A	4.814394	5.139363	0.094235	1.48E-05	0.001292
BCHE	3.865742	3.676101	-0.07257	1.37E-05	0.001292
LPCAT1	6.643714	6.885782	0.051631	1.19E-05	0.001292
CXCL2	5.213695	5.660461	0.118614	1.18E-05	0.001292
NEU4	5.918281	5.620905	-0.07438	1.29E-06	0.000279
CXCL1	6.007135	6.917049	0.20348	8.34E-07	0.000271
CYP2R1	5.847652	5.711935	-0.03388	5.27E-07	0.000271
